# Gut-Derived Metabolomic Biomarkers as Mediators of the Inflammatory Pathway in Early Diabetic Kidney Disease

**DOI:** 10.3390/ijms262411776

**Published:** 2025-12-05

**Authors:** Lavinia Marcu, Carmen Socaciu, Andreea Iulia Socaciu, Adrian Vlad, Florica Gadalean, Flaviu Bob, Oana Milas, Octavian Marius Cretu, Anca Suteanu, Mihaela Glavan, Silvia Ienciu, Maria Mogos, Dragos Catalin Jianu, Sorin Ursoniu, Victor Dumitrascu, Daliborca Vlad, Roxana Popescu, Ligia Petrica

**Affiliations:** 1 Department of Internal Medicine II, Division of Nephrology, “Victor Babes” University of Medicine and Pharmacy, Eftimie Murgu Sq. No. 2, 300041 Timisoara, Romania; lavinia.balint@umft.ro (L.M.); gadalean.florica@umft.ro (F.G.); bob.flaviu@umft.ro (F.B.); milas.oana@umft.ro (O.M.); patruica.mihaela@umft.ro (M.G.); silvia-ioana.ienciu@umft.ro (S.I.); maria.mogos@umft.ro (M.M.); petrica.ligia@umft.ro (L.P.); 2 Centre for Molecular Research in Nephrology and Vascular Disease, Faculty of Medicine, “Victor Babes” University of Medicine and Pharmacy, Eftimie Murgu Sq. No. 2, 300041 Timisoara, Romania; carmen.socaciu@usamvcluj.ro (C.S.); vlad.adrian@umft.ro (A.V.); jianu.dragos@umft.ro (D.C.J.); sursoniu@umft.ro (S.U.); dumitrascu.victor@umft.ro (V.D.); vlad.daliborca@umft.ro (D.V.); popescu.roxana@umft.ro (R.P.); 3 County Emergency Hospital Timisoara, 300723 Timisoara, Romania; 4 Research Center for Applied Biotechnology and Molecular Therapy Biodiatech, SC Proplanta, Trifoiului 12G, 400478 Cluj-Napoca, Romania; 5 Department of Occupational Health, University of Medicine and Pharmacy “Iuliu Haţieganu”, Victor Babes 8, 400347 Cluj-Napoca, Romania; andreeaiso@gmail.com; 6 Department of Internal Medicine II, Division of Diabetes and Metabolic Diseases, “Victor Babes” University of Medicine and Pharmacy, Eftimie Murgu Sq. No. 2, 300041 Timisoara, Romania; 7 Department of Surgery I, Division of Surgical Semiology I, “Victor Babes” University of Medicine and Pharmacy, Eftimie Murgu Sq. No. 2, 300041 Timisoara, Romania; cretu.marius@umft.ro; 8 Emergency Clinical Municipal Hospital Timisoara, 300041 Timisoara, Romania; 9 Department of Neurosciences, Division of Neurology, “Victor Babes” University of Medicine and Pharmacy, Eftimie Murgu Sq. No. 2, 300041 Timisoara, Romania; 10 Centre for Cognitive Research in Neuropsychiatric Pathology (Neuropsy-Cog), Faculty of Medicine, “Victor Babes” University of Medicine and Pharmacy, Eftimie Murgu Sq. No. 2, 300041 Timisoara, Romania; 11 Department of Functional Sciences III, Division of Public Health and History of Medicine, “Victor Babes” University of Medicine and Pharmacy, Eftimie Murgu Sq. No. 2, 300041 Timisoara, Romania; 12 Centre for Translational Research and Systems Medicine, Faculty of Medicine, “Victor Babes” University of Medicine and Pharmacy, Eftimie, Murgu Sq. No. 2, 300041 Timisoara, Romania; 13 Department of Biochemistry and Pharmacology IV, Division of Pharmacology, “Victor Babes” University of Medicine and Pharmacy, No. 2, Eftimie Murgu Sq., 300041 Timisoara, Romania; 14 Department of Microscopic Morphology II, Division of Cell and Molecular Biology II, “Victor Babes” University of Medicine and Pharmacy, No. 2, Eftimie Murgu Sq., 300041 Timisoara, Romania

**Keywords:** early DKD, gut-derived metabolite biomarkers, inflammation biomarkers, metabolomic investigation

## Abstract

Diabetic kidney disease (DKD) is a major complication of type 2 diabetes mellitus (T2DM) and a leading cause of morbidity and mortality. Both metabolic and inflammatory pathways have emerged as potential sources of biomarkers that may improve DKD detection and treatment. This study investigated the relationship between gut-derived metabolites, such as acylcarnitines (ACs), uremic toxins (UTs), polyol pathway intermediates (PIs), and amino acid derivatives (AADs), and renal inflammation markers, detected in serum and urine. It included 20 healthy controls and 90 patients with T2DM, divided into normoalbuminuria, microalbuminuria, and macroalbuminuria. Serum and urine metabolites were analyzed using untargeted and targeted metabolomic assessments, whereas inflammatory markers were quantified using the ELISA technique. Statistical analysis consisted of descriptive statistics followed by univariable and multivariable linear regression analyses. Our findings revealed that serum AADs contribute to renal fibrosis progression, whereas urinary AADs indicate impaired tubular reabsorption in inflammatory conditions. Additionally, UTs and PIs are linked to inflammatory processes mediated by TNF-α but not by early renal fibrosis, whereas serum ACs appear to modulate immune responses, exerting pro-inflammatory and cytotoxic effects on tubular epithelial cells in early DKD. Thus, the metabolic and inflammatory pathways are tightly interconnected and synergistically contribute to the pathogenesis of early DKD.

## 1. Introduction

Diabetes mellitus is a condition that affects half a billion individuals worldwide and leads to severe complications, among which DKD is the most common and most costly to manage in the long term. Up to 30% of patients suffering from T2DM are prone to develop DKD secondary to a decreased estimated glomerular filtration rate (eGFR), whereas 50% of DKD patients will develop albuminuria [1]. Alarming statistical reports demonstrate that half of the patients with DKD will ultimately require dialysis as a result of progression to end-stage renal disease, leading to an increased mortality burden [2].

From a clinical perspective, DKD is characterized as an insidious and progressive condition, often developing asymptomatically over time [3,4]. The traditional DKD diagnosis involves a sustained decline in eGFR or a persistent increase in albuminuria for at least three months [5]. However, by the time these conventional markers become detectable, substantial renal damage has already occurred. As a result, the progression of DKD is exceedingly difficult to halt once the diagnosis is established. This temporal delay highlights a critical limitation of the current diagnostic tools, as they tend to reflect later stages of disease progression [6,7].

The early pathogenesis of DKD is characterized by the activation of metabolic, inflammatory, and hemodynamic pathways [8]. The inflammatory pathway plays a crucial role in DKD development. Persistent hyperglycemia and subsequent hyperfiltration determine the activation of immune cells (macrophages, monocytes, lymphocytes). These cells infiltrate the kidney and increase the synthesis of cytokines and chemokines, which attract other pro-inflammatory cells in an exponential manner [9]. Tumor necrosis factor α (TNF-α) is a cytokine responsible for endothelial cell injury, increased glomerular basement membrane permeability, and subsequent albuminuria [10], whereas transforming growth factor β (TGF-β) determines epithelial-to-mesenchymal transition, extracellular matrix accumulation, and renal fibrosis [11].

Interleukins (ILs), a subset of cytokines, are activators of the immune and inflammatory systemic responses and mediators of intercellular signaling. The constellation of ILs synthesized in T2DM determines renal damage at various levels. Interleukin 6 (IL-6) has multiple phenotypic expressions and has a substantial impact on renal cells: it induces apoptosis and fibrosis in podocytes and mesangial, tubular epithelial, and endothelial cells [12]. Interleukin 8 (IL-8) was found, by our team, in previous studies to be a determinant of podocyte toxicity and proximal tubule dysfunction [13]. While IL-17 is considered a marker of renal disease severity, IL-18 correlates with proximal tubular dysfunction and is a determinant of endothelial cell apoptosis [14]. Unlike the aforementioned ILs, IL-10 has anti-inflammatory effects and was found to decrease renal inflammation and fibrosis and to cease mesangial cell proliferation [15].

Metabolomics is an advanced analytical approach, which allows for the detailed profiling of an individual’s metabolic state and offers insights into how persistent hyperglycemia in T2DM disrupts homeostasis across multiple biochemical pathways [16,17]. Additionally, gut microbiota, a key component of the host’s metabolome, is notably altered under chronic hyperglycemic conditions, resulting in intestinal dysbiosis and playing a significant role in T2DM complication development [18].

Recent studies combining metabolomics with bioinformatic pathway analysis have revealed disruptions in amino acid, purine/nucleotide, and bile acid metabolism, along with the downregulation of renal tubular transporters in DKD patients [19]. Transcriptomic analyses using network-based approaches in DKD models further emphasize altered metabolic pathways, linking metabolic dysregulation to structural renal damage [20]. Additional research highlights the interconnections between the metabolic and the inflammatory pathways, underscoring their potential in improving early diagnosis [21]. Collectively, these findings, the metabolic alterations, uremic toxin accumulation, and changes in metabolism-related gene expression, support a mechanistic crosstalk among pathogenic pathways in DKD.

In our extended research on biomarkers in patients with T2DM, we first carried out an untargeted metabolomic investigation on blood and urine samples, using both multivariable and univariable analyses. This untargeted approach indicated the main metabolic pathways involved in T2DM and early DKD pathogenesis, and the results were presented in a previous publication [22]. In our second study, we performed a targeted and quantitative analysis of seven serum and seven urine metabolites derived from previously investigated metabolic pathways (tyrosine, tryptophan, phenylalanine) and additional metabolic pathways. These metabolites showed strong biomarker potential and clearly distinguished patients with early DKD and normal-to-mildly increased albuminuria from controls and from those with moderate or severe albuminuria [23]. In our third investigation, we examined the relationship between the previously quantified metabolites and markers of renal endothelial, podocyte, and proximal tubule damage, as well as cerebrovascular damage indices in the same group of patients. Our findings highlighted the involvement of these metabolites in T2DM complications, including the early pathogenic changes in DKD and cerebrovascular microangiopathy [24].

This investigation introduces a novel approach by exploring the complex relationship between two main pathogenic pathways in DKD, the metabolic and inflammatory pathways. The present investigation aims to identify the potential associations between the metabolites detected in serum and urine, previously characterized by untargeted ultra-high-performance liquid chromatography coupled with electrospray ionization–quadrupole–time-of-flight mass spectrometry (UHPLC-QTOF-ESI^+^-MS) and quantified through targeted analyses, and corresponding inflammatory biomarkers (interleukins, TNF-α, TGF-β) identified in the serum and urine of patients suffering from T2DM and DKD.

Studying these alterations is particularly important for a deeper understanding of the complex pathogenic mechanisms underlying DKD. Studies on gut microbiota-derived compounds and their fluctuations may change our comprehension of T2DM and DKD pathophysiology.

## 2. Results

### 2.1. Clinical and Demographic Parameters

The parameters resulting from clinical examination, the patients’ medical history, and usual blood and urine tests are displayed in Table 1. Moreover, Table 1 contains the parameters obtained from UHPLC-QTOF-ESI^+^-MS and an ELISA assay. All the parameters are displayed as medians and interquartile ranges. Group C was differentiated by group P by applying one-way analysis of variance (ANOVA) coupled with Bonferroni correction analysis, the Kruskal–Wallis test, and a chi-squared test. The comparison between the subgroups of patients (P1 vs. C, P1 vs. P2, P2 vs. P3) was realized by several tests, such as Student’s *t*-test, the chi-squared test, and Mann–Whitney test.

The descriptive statistical analysis shown in Table 1 reveals a strong differentiation between the selected subgroups (C vs. P1, P1 vs. P2, P2 vs. P3, respectively, C vs. P1 vs. P2 vs. P3) of all serum and urine inflammatory markers. Additionally, it may be observed that only certain metabolites are significant when comparing C vs. P1 and P1 vs. P2, such as sIS, uIS, sArg, uArg, sBCA, uBCA, sSorb, and uPCS.

### 2.2. Association Between Circulating Metabolites and Systemic Inflammatory Biomarkers

#### 2.2.1. Univariable Linear Regression Analysis

According to Supplementary Table S1, sArg exhibited a strong negative correlation with both sTNF-α and sIL-8, as well as a modest positive correlation with sIL-10 and a negative correlation with sIL-18. Additionally, sSorb demonstrated a strong positive correlation with both sTNF-α and sIL-6. In contrast, uremic toxins such as sHA and sIS did not show significant associations with serum inflammatory markers in univariable linear regression analysis.

With regard to urinary markers, as can be seen in Supplementary Table S2, the most prominent associations were observed between uTNF-α and both uArg and uHA. Moreover, uIS, uLAC, uBCA, and uPCS were all strongly positively correlated with uTNF-α, uIL-6, and uFABP. Additionally, uLAC displayed a weaker but notable correlation with uIL-12.

#### 2.2.2. Multivariable Linear Regression Analysis

Serum multivariable linear regression analysis is displayed in Table 2, and it shows that all serum metabolites are correlated with sTNF-α in a positive manner (except sArg, which correlated in a negative manner). Moreover, all metabolites but sSorb correlate with sTGF-β (sArg and sHA display a positive correlation, unlike sLAC and sBCA, which correlate negatively). Going further, the multivariable analysis exhibits a negative correlation of sArg vs. sIL-8, sLAC vs. sIL-18, sSorb vs. IL-17, and sHA vs. sIL-17, respectively.

The results of the urine multivariable analysis, presented in Table 3, demonstrate multiple statistically significant correlations between urine metabolites and inflammatory markers, as indicated by the *p* values and R^2^ values. Consistent with the serum analysis, all urinary metabolites exhibit correlations with uTNF-α, uTGF-β, and uACR. Extending these observations, uIL-6 shows a positive correlation with uIS and uBCA. Conversely, uIL-18 displays a negative correlation with uIS, while uIL-8 is negatively correlated with both uArg and uBCA.

### 2.3. Metabolite Origin

Information from the scientific literature regarding metabolite provenance, excretion pathways, and the references supporting these data are displayed in Table 4.

## 3. Discussion

This study was performed on blood and urine samples of DKD patients suffering from T2DM, which were analyzed by metabolomic techniques such as UHPLC-QTOF-ESI^+^-MS, in order to obtain the metabolites, and by the ELISA method, in order to achieve the markers of inflammation. The subsequent statistical analyses (univariable and multivariable regression analyses) revealed interesting associations between the metabolites and the inflammatory markers. These findings bring a novelty to the field of T2DM and DKD research by providing a set of key biomarkers specific to DKD onset that may act as targets for new therapies in the future.

### 3.1. Serum Arginine Is a Biomarker of Early Renal Fibrosis in DKD via TGF- β Production Whereas Urine Arginine Associates with Early Inflammation in the Tubulo-Interstitial Compartment

In the current study, Table 1 shows how sArg and uArg differentiate group C vs. P1 and P1 vs. P2, indicating the biomarker role, in serum and urine, of this AA in early DKD. Further on, in the multivariable regression analysis, indicated in Table 2, sArg correlates negatively with sTNF-α and sIL-8, and positively with sTGF-β. Conversely, by shifting the focus to the urinary dynamics of this metabolite, we may observe that its levels gradually increase from controls to P1–P3 subgroups, as presented in Table 1. Additionally, the multivariable regression analysis, indicated by Table 3, shows the positive correlation of uArg with uTNF-α and uACR and its negative correlation with uIL-8 and uTGF-β.

L-arginine is an AA derivative whose metabolism occurs primarily in the gastrointestinal tract, yielding either nitric oxide or dimethylarginines in the context of DKD. These metabolic pathways are closely interconnected, with nitric oxide synthesis promoting further vasodilation, whereas the production of asymmetric dimethylarginine (ADMA) contributes to endothelial dysfunction [32,33,34]. In the normal state, consecutively to its intestinal absorption, L-arginine enters the blood flow and becomes subjected to glomerular filtration and proximal tubular reabsorption [35].

Elevated sTGF-β levels have been associated with increased ADMA production, which in turn has been implicated in the reorganization of the podocyte actin cytoskeleton. TGF-β functions as a pro-fibrotic cytokine, modulating both the synthesis and degradation of extracellular matrix components [36]. Serum TNF-α is a pro-inflammatory cytokine that stimulates the production of reactive oxygen species and has been shown to increase endothelial permeability to albumin, whereas uTNF-α is indicative of renal tubulo-interstitial inflammation [37]. In parallel, sIL-8 acts as a promoter of oxidative stress and is involved in mesangial cell proliferation [38], whereas uIL-8 was found to be indicative of early renal damage in DKD [14].

The observed correlation of sArg and sTGF-β (Table 2) suggests a potential involvement of sArg in renal fibrosis and actin cytoskeleton remodeling via ADMA-mediated mechanisms. Conversely, the negative correlations between sArg and both sTNF-α and sIL-8 (Table 2) indicate that sArg may exert protective and anti-inflammatory effects, possibly by favoring NO synthesis over ADMA production in early DKD. Moreover, the multivariable regression analysis did not indicate a correlation of sArg with eGFR (Table 2), unlike uArg, which correlated with uTNF-α and uACR (Table 3). These results may suggest that Arg is increasingly excreted as DKD progresses, a process potentially driven by albumin loss, which is indicative of impaired AA reabsorption in the proximal tubule, under conditions of inflammatory stress.

### 3.2. Uremic Toxins’ Involvement in the Inflammatory Pathway in DKD

#### 3.2.1. Hippuric Acid in Serum and Urine Reflects a Predominant Inflammatory State in Early DKD, Rather than Serving as a Marker of Renal Fibrosis

Our study confirms the potential role of sHA as a biomarker of early DKD, according to Table 1. Table 2 shows its correlations with sTNF-α and eGFR in a positive manner, and with sTGF-β and sIL-17 in a negative manner. Moreover, in urine, uHA is associated positively with uTNF-α, uACR, and IL-10 and negatively with uTGF-β, according to Table 3.

HA is a uremic toxin that results from the breakdown of dietary phenylalanine and polyphenols under the gut microbiota’s action [39]. Its metabolism is highly dependent on the hepatic and renal mitochondrial population and is mainly excreted via the renal route. HA was previously proven to be a renal pro-fibrotic and pro-oxidative metabolite, and ultimately, a promoter of chronic kidney disease development [40].

TNF-α is a strong predictor of eGFR decline by enhancing renal inflammation and oxidative stress, endothelial cell damage, and tubulo-interstitial injury. In addition, sTGF-β is known to be a marker of renal fibrosis, showing progressively increased levels as eGFR declines, whereas sIL-17 indicates chronic inflammation and an increased state of autoimmunity, being linked to podocyte damage [41].

The observed positive correlation between sHA, sTNF-α, and eGFR (Table 2) shows that increased levels of sHA are associated with the activation of the inflammatory pathway and reflect dysregulated clearance of this metabolite, even before advanced renal disease occurs. Also, the negative association of sHA with sTGF-β and sIL-17 indicates that in early DKD stages, the inflammatory state is dominant via increased sTNF-α. This finding points to a possible shift from an inflammatory to a fibrotic state as DKD progresses.

Regarding the urine findings, we may conclude that the positive association of uHA with uTNF-α and uACR (Table 3) is reflective of renal tubular damage and local inflammation. As IL-10 is well known to be an anti-inflammatory cytokine [15], its positive correlation with uHA may show that a compensatory regulatory response is underway in early DKD. On the other hand, the negative correlation with uTGF-β (Table 3) still indicates that the pro-fibrotic signaling is subdominant in the early stages of renal disease.

#### 3.2.2. Indoxyl Sulfate

The dynamic of uIS, in our investigation, is displayed in Table 3 and indicates the correlation of this metabolite with uTNF-α, uIL-6, and uACR. Inversely, uTGF-β and uIL-18 are associated in a negative manner with uIS.

Dietary tryptophan is metabolized by gut microbiota into indole, which is subsequently sulfated in the liver to form IS [42]. This metabolite is a renal-excreted protein-bound uremic toxin and was found to increase in certain patient populations, such as those suffering from T2DM and chronic kidney disease, secondary to reduced eGFR or increased production in the context of gut dysbiosis [43]. From a pathogenic renal perspective, IS exerts harmful effects across all renal structures, promoting oxidative stress and fibrosis in tubular cells and podocytes, while also amplifying inflammatory responses that impair renal endothelial cell function [44].

The positive association of uIS with uTNF-α, uIL-6, and uACR (Table 3) indicates that higher levels of uIS are associated with higher renal tubular exposure to this metabolite or its reduced renal clearance. This may determine an increased production of uTNF-α and uIL-6 by renal tubular cells, indicating the role of uIS in amplifying inflammation in early DKD stages. The negative correlation of uIS is contradictory to the literature data findings, in which uTGF-β and uIL-18 are reported to be positively correlated with this metabolite [45]. Our finding is, thus, controversial, and may suggest a stage-specific pathogenic mechanism in which DKD is associated in its early stages with an inflammatory pattern rather than a pro-fibrotic one.

#### 3.2.3. P-Cresyl Sulfate

Similarly to uIS, uPCS is associated in a positive manner with uTNF-α and uACR, but not with uIL-6, and in a negative manner with uTGF-β, according to Table 3.

P-Cresyl sulfate is a gut-derived uremic toxin formed through the hepatic sulfation of phenolic compounds. These phenolic precursors originate from the microbial fermentation of aromatic amino acids, primarily tyrosine and phenylalanine, under the action of intestinal microbiota [28]. Under conditions of gut dysbiosis, such as those associated with hyperglycemia, this metabolic pathway becomes dysregulated, leading to increased production and accumulation of pCS. Experimental data reveals that uPCS induces oxidative stress and stimulates pro-inflammatory and pro-fibrotic signaling in renal tubular cells, thereby contributing to kidney damage [46]. In DKD, elevated concentrations of uPCS have been linked to adverse renal outcomes, including a more rapid decline in eGFR and increased albuminuria [47].

In our findings, uPCS follows the same trend of correlations as uIS does. We may postulate that uPCS’s association with uTNF-α and uACR indicates renal tubular injury and inflammation, which determine glomerular basement and podocyte damage, and subsequent progressive albumin loss. Shifting the attention to the uPCS–uTGF-β correlation, our findings are debatable, as they are not in agreement with recent experimental findings in which these molecules follow the same trend [48]. As mentioned above, when focusing on IS data, we may link these findings to the disease phase or early inflammatory pathway activation.

### 3.3. Serum Sorbitol Exerts Nephrotoxicity and Contributes to DKD Development

Our data indicates the biomarker potential of sSorb in early DKD, based on the statistical significance between subgroups presented in Table 1. Further on, multivariable regression analysis reveals a positive correlation between sSorb, sTNF-α, and eGFR and a negative correlation between sSorb and sIL-17, as presented in Table 2.

Sorbitol is a by-product of the polyol pathway that becomes activated under hyperglycemic conditions [30]. The in vitro study of Lagies et al., conducted on cultured cell lines such as podocytes and proximal tubule cells, demonstrated an exacerbation of the polyol pathway and subsequent sorbitol production under hyperglycemic stress [49]. Over time, the nephrotoxic effects of TNF-α have been demonstrated in multiple ways: it contributes to the development of T2DM complications, and it exerts toxic effects on mesangial, endothelial, and glomerular cells [10].

The direct association between circulating sSorb levels and sTNF-α (Table 2) not only reinforces the hypothesis of sorbitol’s contribution to the development of DKD but also demonstrates its involvement in two key pathogenic mechanisms in early disease onset: the metabolic and inflammatory pathways. Thus, we may assume that sorbitol is nephrotoxic and contributes to DKD development by interfering with the inflammatory pathway. Furthermore, our findings validate previous in vitro evidence of polyol pathway-induced nephrotoxicity by providing in vivo data.

### 3.4. Serum and Urine Butenoylcarnitine May Be a Biomarker of Early Renal Inflammation in DKD

Table 1 indicates that sLAC and uLAC could not be considered biomarkers of early DKD as they do not differentiate the subgroups of patients from controls.

Conversely, according to Table 1, sBCA and uBCA express biomarker potential in early DKD. Moreover, Table 2 shows a positive correlation between sBCA, sTNF-α, and sTGF-β, and no correlation with eGFR, while Table 3 points to a positive correlation between uBCA, uTNF-α, uIL-6, and uACR and a negative correlation between uBCA, uTGF-β, and uIL-8.

The kidney ranks second to the heart in terms of mitochondrial abundance, the proximal tubule being the main site of lipid breakdown. This is particularly important, as the β-oxidation process requires the involvement of acylcarnitines as carriers of fatty acids inside the mitochondria [50]. Serum acylcarnitines reflect the serum fatty acid β-oxidation rate, and their high levels are indicative of mitochondrial dysfunction in the course of DKD [51]. Moreover, the dysregulated levels of urinary acylcarnitines were found to be associated with early renal lesions in T2DM [52].

As sTNF-α and sTGF-β are generally opposite forces, with the first one being pro- and the second one exerting anti-inflammatory effects, we may postulate, based on our findings, that sBCA is not only an indicator of mitochondrial dysfunction in DKD but also a promoter of immune cell activation and cell apoptosis via the inflammatory pathway. On the other hand, uIL-8 was found to be linked to worse overall renal outcomes [53]. Thus, the positive association with uBCA indicates this carnitine as a potential urinary biomarker of DKD development, but its involvement in renal outcomes remains to be proven. Moreover, the absence of an association between sBCA and eGFR indicates that its progressively elevated serum levels, during DKD progression, are more likely attributable to increased endogenous production rather than reduced excretion secondary to declining eGFR.

### 3.5. The Limitations and Strengths of This Study

Our investigation has several limitations that should be acknowledged. As a pilot and cross-sectional investigation, metabolite and inflammatory markers’ levels were determined only once and this precludes the establishment of causal relationships. Also, the number of participants in each subgroup was relatively small, which may reduce the statistical power and limit the generalizability of the findings. Detailed history regarding the duration of chronic treatment and drug doses was not available for all participants, and this may interfere with the levels of the identified metabolites. Since stool samples were unavailable, the intestinal origin of the detected metabolites was only considered based on the data we found in the scientific literature.

Despite its limitations, our study also has its strengths. To the best of our knowledge, this is the first metabolomic investigation to integrate and examine the interplay between early pathogenic mechanisms of metabolic and inflammatory pathways in DKD. Our study also indicates that the gut-derived metabolites found may contribute to early DKD development in a phase-dependent manner, being more strongly associated with inflammatory processes rather than with renal fibrosis. These findings may not only contribute to the early diagnosis of DKD but also provide valuable guidance for the development of novel therapeutic targets aimed at mitigating inflammation and thereby preventing disease onset or slowing disease progression.

## 4. Materials and Methods

### 4.1. Ethical Standards and the Selection of Study Participants

From July 2021 to April 2022, 130 individuals with T2DM were screened at the County Emergency Hospital’s Nephrology and Diabetes and Metabolic Diseases Departments in Timisoara, Romania. This study was conceptualized to display a pilot and a cross-sectional design. Ultimately, there were 90 T2DM patients (P group) and 20 individuals in good health (C group) included. According to the KDIGO and ADA Consensus, the disease-related group (P) was divided into three subgroups based on uACR: P1 (normal to mildly increased albuminuria/normoalbuminuria), P2 (moderately increased albuminuria/microalbuminuria), and P3 (severely increased albuminuria/macroalbuminuria). This study comprised participants diagnosed with T2DM for at least 5 years, without a history or clinical manifestations of cerebrovascular maladies, and an HbA1c level of less than 10%. The investigation excluded patients with HbA1c levels above 10%, other glomerulonephritis, acute or chronic infections (particularly urinary tract infections), malignancies, autoimmune, or mental illnesses. T2DM patients received therapy with oral antidiabetic medications, insulin, angiotensin 2-converting enzyme inhibitors/receptor blockers, and statins. This investigation was approved by the Ethics Committees at Victor Babes University of Medicine and Pharmacy, Timisoara (29/30 June 2021) and the County Emergency Hospital, Timisoara (220/18 January 2021). All subjects provided informed consent in writing, and the study followed the Declaration of Helsinki requirements.

### 4.2. The Preparation of Samples

The blood was drawn by venipuncture in an anticoagulant-free, sterile vacutainer, whereas the urine was collected using a sterile vial. The following steps were taken to prepare the samples for UHPLCQTOF-ESI^+^-MS analysis: 0.8 mL mix of pure HPLC-grade methanol and acetonitrile (2:1 *v*/*v*) was added for each 0.2 mL of urine and 0.2 mL of serum. The samples were subjected to 5 min of ultrasonication, followed by vortexing, and then stored at 20 °C for 24 h to allow the precipitation of proteins. Furthermore, the supernatant was centrifuged to be prepared for filtration through 0.2 μm Nylon filters. It was then transferred to micro-vials and deposited into the autosampler of the UHPLC.

### 4.3. Evaluation Techniques and Further Analysis of the Samples

#### 4.3.1. Metabolomic Assessment

In order to carry out the metabolomic assessment, a Thermo Fisher Scientific UHPLC-QTOF-ESI+-MS Ultimate 3000 (Pittsburg, PA, USA) instrument was utilized. This instrument included a quaternary pump, Dionex delivery system, and MS detection technology with MaXis Impact (Bruker Daltonics, Billerica, MA, USA). Additional information regarding the procedures that were utilized for the analysis of untargeted UHPLCQTOF-ESI+-MS is found in our first investigation.

The metabolites were quantified using the following reagents and chemicals: Sigma-Aldrich (Burlington, MA, USA) supplied PLC-grade formic acid, whereas Fisher Scientific (Pittsburgh, PA, USA) provided HPLC/MS-grade formic acid and acetonitrile. The utilized pure standardized biomarkers were represented by arginine from amino acid standard H (product #20088, Thermo Scientific) MW = 174; acetyl-Lcarnitine hydrochloride (J6153606; Alfa Aesar by Thermo Fisher) MW = 203; asymmetric dimethyl-L-arginine _ 95% (HPLC) CAS (Thermo Scientific) 30315-93-6 (Sigma Aldrich) MW = 202.25; indoxyl sulfate potassium salt, 97%, (A1707901; Alfa Aesar by Thermo Fisher) MW = 213; hippuric acid, 98%, (A1269022; Alfa Aesar by Thermo Fisher) MW = 179; Sorbitol, >98% product S1876 Sigma-Aldrich Chemie GmbH, MW = 182; creatinine > 98% product C4255, Sigma-Aldrich Chemie GmbH, MW = 113 and p-Cresyl sulfate, >98%, product 29,504 Cayman Chemical, US, MW = 188.

Doxorubicin hydrochloride (MW = 580) (injectable, 2 mg/mL, Sun Pharmaceutical Industries) served as the internal standard. The ultra-high-purity water was generated using the Millipore-Q Water Purification System (Millipore, Germany). LC–MS-grade methanol, acetonitrile, and formic acid were acquired from Fisher Scientific (Loughborough, UK). Ultra-pure water was obtained by a Milli-Q water purification system (Millipore, Milford, MA, USA). The instruments utilized in this work comprised a vortex mixer, a Minicentrifuge Eppendorf (Thermo Fisher Scientific, USA), and a UPLC-Q-TOF/MS (Bruker GmbH, Bremen, Germany).

#### 4.3.2. ELISA Assessments

The serum and urine samples were frozen at −80 °C and subsequently thawed prior to analysis. Urinary biomarkers were assessed in the early morning sample of urine and reported according to the urinary creatinine. The inflammation biomarkers examined were evaluated using the ELISA technique, specifically as follows: tumor necrosis factor alpha (TNFα, Catalog No. E-EL-H0109 Elabscience, Houston, TX, USA; sensitivity—4.69 pg/mL, detection range—7.81–500 pg/mL, CV < 10%); interleukins [IL-17A (Catalog No. E-EL-H0105 Elabscience, Houston, TX, USA; sensitivity—18.75 pg/mL, detection range—31.25–2000 pg/mL, CV < 10%), IL-18 (Catalog No. E-EL-H0253 Elabscience, Houston, TX, USA; sensitivity—9.38 pg/mL, detection range—15.63–1000 pg/mL, CV < 10%), and IL-10 (Catalog No. E-EL-H6154 Elabscience, Houston, TX, USA; sensitivity—0.94 pg/mL, detection range—1.56–100 pg/mL, CV < 10%)]. All serum and urine samples were evaluated according to the manufacturer’s guidelines.

#### 4.3.3. Statistical Analysis

The statistical analyses were carried out according to the methodological criteria used for exploratory case series studies. Data regarding the clinical and the biological parameters are reported as medians and interquartile ranges for skewed variables. Comparisons between two subgroups were performed using the Mann–Whitney U test, while differences among four subgroups were assessed with the Kruskal–Wallis test, based on the data distribution. To examine the statistical significance of the connection between metabolites and markers of inflammation, regression analysis was conducted. Univariable regression analyses were performed to investigate the relationships among continuous variables across all four groups, including data obtained from healthy controls and patients with normoalbuminuric, microalbuminuric, or macroalbuminuric profiles. Variables expressing significance in univariable analyses were subsequently introduced in the multivariable regression models. Multicollinearity was assessed using the variance inflation factor (VIF), with values exceeding 10 considered indicative of potential collinearity. Statistical significance was defined as *p* < 0.05. All analyses were conducted using Stata 18 (StataCorp, College Station, TX, USA).

## 5. Conclusions

In conclusion, our study demonstrates the connection of a panel of metabolomic biomarkers with markers of inflammation in the early stages of DKD. Specifically, sArg may play a role in the renal fibrotic process, whereas uArg may indicate impaired renal tubular reabsorption under pro-inflammatory conditions. Sorb and uremic toxins, such as HA, IS, PCS, appear to be associated primarily with enhanced inflammation rather than with renal fibrosis in early DKD. Additionally, BCA may serve as an early indicator of DKD development through its involvement in the inflammatory pathway.

## Figures and Tables

**Table 1:** Overall parameters: demographic data, conventional biological parameters, urine and serum metabolites, urine and serum inflammatory markers, and correlations among patient subgroups.

Parameter	20 Healthy Controls	30 Patients With Normal to Mildly Increased Albuminuria	30 Patients with Moderately Increased Albuminuria	30 Patients with Severely Increased Albuminuria
Clinical features				
Male (number, %)	12 (60%)	14 (46.66%)	13 (43.33%)	15 (50%)
Age (years)	58.85 (7.25)	68.41 (4.98)	68.65 (4.91)	68.84 (4.98)
Body mass index	24.75 (4.39) ^●ↂ^	30.07 (4.54)	31.51 (4.01)	30.98 (5.28)
Duration of DM (years)	0 ^●ↂ^	9.6 (3.99)	9.7 (3.99)	12.78 (3.35)
DR (number, %)	0 ^†^	5 (16.66%)	10 (33%) ^♣^	20 (66.66)
DN (number, %)	0 ^●^	3 (10%)	9 (30%)	16 (53.33)
Common blood and urine parameters				
eGFR (mL/min/1.73 m^2^)	97.93 (11.71) ^●ↂ^	90.42 (18.10) ^▲^	67.80 (5.44) ^♣^	49.53 (9.4)
uACR (mg/g)	5 (0.23) ^●ↂ^	7.38 (3.22) ^▲^	45.52 (47.08) ^♣^	319.86 (585.8)
Triglycerides (mg/dL)	111.05 (20.73) ^†ↂ^	139.65 (51.1)	172.63 (93.63) ^■^	227.17 (107.55)
Cholesterol (mg/dL)	132.5 (24.62) ^†ↂ^	163.62 (54.39)	166.8 (57.7) ^■^	199.5 (48.1)
HbA1c (%)	4.98 (0.23) ^●ↂ^	5 (0.23) ^♦^	6.42 (1.29) ^♣^	7.15 (1.60)
Serum and urine metabolites				
sArg (μM)	50.03 (10.02) ^†ↂ^	44 (10.18) ^♦^	38.4 (6.5)	38.91 (7.67)
sHA (μM)	24.33 (2.35) ^†⁑^	22.71 (1.24)	22.39 (1.72)	20.79 (5.1)
sIS (μM)	5.06 (0.45) ^●ↂ^	5.14 (0.47) ^♦^	6.51 (5.07) ^♣^	6.63 (0.6)
sLAC (μM)	5.52 (2.13)	5.72 (2.07)	5.47 (1.74)	5.73 (1.6)
sBCA (μM)	2.3 (0.1) ^ↂ^	2.25 (0.13) ^▲^	2.61 (0.37)	2.51 (0.55)
sSorb (μM)	2.54 (0.46) ^†ↂ^	2.25 (0.14) ^▲^	2.67 (0.3)	2.59 (0.33)
uArg/uCr (μM/μM)	5.26 (1.72)	6.08 (2.86) ^♦^	4.84 (3.22)	5.63 (2.51)
uLAC/uCr (μM/μM)	0.31 (0.13)	0.40 (0.26)	0.42 (0.31)	0.54 (0.54)
uBCA/uCr (μM/μM)	0.24 (0.11) ^●ↂ^	0.45 (0.23)	0.45 (0.32)	0.53 (0.26)
uHA/uCr (μM/μM)	52.55 (29.36)	54.66 (2.85)	57.75 (72.7)	55.62 (29.86)
uIS/uCr (μM/μM)	0.82 (0.37) ^ↂ^	2.07 (1.09)	1.99 (1.5) ^■^	2.44 (1.21)
uPCS/uCr (μM/μM)	3.39 (1.62) ^†⁑^	5.69 (5.14)	5.65 (3.54)	7.64 (4.5)
Parameters of inflammation from serum				
sTNF-α (pg/mL)	15.82 (4.91) ^●ↂ^	27.46 (9.83) ^▲^	54.23 (14.51) ^♣^	113.37 (37.03)
sTGF-β (pg/mL)	0.22 (0.05) ^●ↂ^	0.45 (0.21) ^▲^	3.25 (1.11) ^♣^	8.96 (0.70)
sIL-6 (pg/mL)	3.80 (0.88) ^●ↂ^	6.54 (1.77) ^▲^	15.03 (5.33) ^♣^	40.19 (19.20)
sIL-8 (pg/mL)	38.93 (23.41) ^●ↂ^	108.09 (30.03) ^▲^	184.55 (19.97) ^♣^	257.64 (39.28)
sIL-10 (pg/mL)	20.81 (4.06) ^●ↂ^	13.97 (2.15) ^▲^	11.22 (1.08) ^■^	9.44 (2.03)
sIL-12 (pg/mL)	25.21 (3.70) ^●ↂ^	126.17 (47.97) ^▲^	178.21 (50.35) ^♣^	308.88 (94.82)
sIL-17 (pg/mL)	81.38 (16.97) ^●ↂ^	152.54 (26.19) ^▲^	274.15 (33.85) ^♣^	460.31 (75.53)
sIL-18 (pg/mL)	84.25 (21.86) ^●ↂ^	118.64 (39.41) ^▲^	154.09 (42.17) ^♣^	218.66 (78.52)
Parameters of inflammation from urine				
uTNF-α (pg/g)	13.31 (3.68) ^●ↂ^	21.46 (7.84) ^▲^	40.97 (15.18) ^♣^	64.41 (21.73)
uTGF-β (pg/g)	0.19 (0.01) ^●ↂ^	0.29 (0.07) ^▲^	0.79 (0.30) ^♣^	4.97 (1.32)
uIL-6 (pg/g)	2.33 (0.68) ^●ↂ^	5.06 (1.59) ^▲^	8.04 (2.05) ^♣^	15.83 (3.77)
uIL-8 (pg/g)	8.72 (0.61) ^●ↂ^	30.10 (9.55) ^▲^	102.36 (27.17) ^♣^	159.64 (36.49)
uIL-10 (pg/g)	15.32 (2.10) ^●ↂ^	8.89 (2.48) ^▲^	5.73 (1.28) ^■^	4.79 (1.41)
uIL-12 (pg/g)	25.21 (2.14) ^●ↂ^	126.17 (19.58) ^▲^	178.21 (26.37) ^♣^	308.88 (44.57)
uIL-17 (pg/g)	53.07 (11.55) ^●ↂ^	120.70 (30.45) ^▲^	241.07 (57.02) ^♣^	408.45 (67.86)
uIL-18 (pg/g)	29.45 (13.18) ^†ↂ^	46.43 (22.13) ^♦^	95.02 (31.97) ^♣^	142.85 (50.90)

Data are expressed as medians and interquartile range; statistical significance was assessed using Student’s *t*-test, chi-square test, or Mann–Whitney U test, depending on data type and distribution. Significance of C vs. P1: ^●^ *p* < 0.001; ^†^ *p*: 0.001–0.05; significance of P1 vs. P2: ^▲^ *p* <0.001; ^♦^ *p*: 0.001–0.05; significance of P2 vs. P3: ^♣^ *p* < 0.001; ^■^ *p*: 0.001–0.05; significance of C vs. P1 vs. P2 vs. P3: ^ↂ^ *p* < 0.001; ^⁑^ *p*: 0.001–0.05; DM: diabetes mellitus; DR: diabetic retinopathy; DN: diabetic neuropathy; eGFR: estimated glomerular filtration rate; uACR: urinary albumin/creatinine ratio; sArg: serum arginine; sHA: serum hippuric acid; sIL-6-8-10-12-17-18: serum interleukin 6-8-10-12-17-18; sIS: serum indoxyl sulfate; sLAC: serum L-acetylcarnitine; sBCA: serum butenoylcarnitine; sSorb: serum sorbitol; sTGF-β: serum transforming growth factor β; sTNF-α: serum tumor necrosis factor α; HbA1c: glycated hemoglobin; uArg/uCr: urinary arginine-to-creatinine ratio; uHA/uCr: urinary hippuric acid-to-creatinine ratio; uIL-6-8-10-12-17-18: urinary interleukin 6-8-10-12-17-18; uIS/uCr: urinary indoxyl sulfate-to-creatinine ratio; uLAC/uCr: urinary L-acetylcarnitine; uBCA/uCr: urinary butenoylcarnitine-to-creatinine ratio; uPCS/uCr: urinary p-cresyl sulfate-to-creatinine ratio.

**Table 2:** Multivariable linear regression analysis of serum metabolites and inflammatory markers.

Dependent Variable	Independent Variables	Coef β	*p*	95% CI	Prob > F	R^2^
sArg	sTNF-α	−0.1967	<0.0001	−0.27 to −0.11	<0.0001	0.4321
	sTGF-β	2.7911	<0.0001	1.08 to 3.77		
	sIL-8	−0.0789	<0.0001	−0.11 to −0.04		
sHA	sTNF-α	0.1428	<0.0001	0.11 to 0.16	<0.0001	0.629
	sTGF-β	−0.6029	0.001	−0.94 to −0.26		
	sIL-17	−0.0218	<0.0001	−0.02 to −0.01		
	eGFR	0.0696	0.009	0.01 to 0.12		
sSorb	sTNF-α	0.0096	<0.0001	0.006 to 0.01	<0.0001	0.336
	sIL-17	−0.0009	0.035	−0.001 to −0.00006		
	eGFR	0.0089	0.014	0.001 to 0.01		
sLAC	sTNF-α	0.0594	<0.0001	0.04 to 0.07	<0.0001	0.325
	sTGF-β	−0.4683	<0.0001	−0.65 to −0.27		
	sIL-18	−0.0067	0.022	−0.012 to −0.001		
sBCA	sTNF-α	0.0128	<0.0001	0.009 to 0.01	<0.0001	0.4704
	sTGF-β	−0.0976	<0.0001	−0.13 to −0.06		

**Table 3:** Multivariable linear regression analysis of urine metabolites and inflammatory markers.

Dependent Variable	Independent Variables	Coef β	*p*	95% CI	Prob > F	R^2^
uArg	uTNF-α	0.0782	<0.0001	0.04 to 0.104	<0.0001	0.3676
	uTGF-β	−0.6002	0.002	−0.97 to −0.22		
	uIL-8	−0.0241	<0.0001	−0.03 to −0.01		
	uACR	0.0032	<0.0001	0.001 to 0.004		
uHA	uTNF-α	1.112	<0.0001	0.61 to 1.61	<0.0001	0.2616
	uTGF-β	−10.8716	0.001	−17.13 to −4.605		
	uIL-10	2.4008	0.04	0.107 to 4.69		
	uACR	0.0286	0.01	0.005 to 0.051		
uIS	uTNF-α	0.0229	0.001	0.01 to 0.03	<0.0001	0.533
	uTGF-β	−0.454	<0.0001	−0.62 to −0.28		
	uIL-6	0.1508	0.002	0.05 to 0.24		
	uIL-18	−0.0055	0.043	−0.01 to −0.0001		
	uACR	0.001	<0.0001	0.0004 to 0.001		
uPCS	uTNF-α	0.0706	0.001	0.03 to 0.11	<0.0001	0.5416
	uTGF-β	−1.541	<0.0001	−2.07 to −1.006		
	uACR	0.007	<0.0001	0.005 to 0.009		
uLAC	uTNF-α	0.0044	0.001	0.001 to 0.007	<0.0001	0.6258
	uTGF-β	−0.1377	<0.0001	−0.17 to −0.102		
	uACR	0.0006	<0.0001	0.0005 to 0.0008		
uBCA	uTNF-α	0.006	<0.0001	0.003 to 0.008	<0.0001	0.5283
	uTGF-β	−0.0686	<0.0001	−0.12 to −0.05		
	uIL-6	0.0242	0.0065	0.006 to 0.04		
	uIL-8	−0.0013	0.024	−0.002 to −0.0001		
	uACR	0.0002	<0.0001	0.0001 to 0.0003		

**Table 4:** Metabolite-related data: origins, excretion routes, and references.

Metabolite	Origin	Excretion Route	Reference
Arginine	Proteolysis of dietary proteins De novo synthesis in the renal–intestinal axis	Renal	[25]
Hippuric acid	Glycine conjugation of benzoic acid in the liver Gut microbial metabolism of phenylalanine	Renal	[26]
Indoxyl sulfate	Metabolism of dietary tryptophan by gut microbiota Hepatic sulfation of indole	Renal	[27]
P-cresyl sulfate	Metabolism of dietary tyrosine and phenylalanine by gut microbiota Hepatic sulfation of p-cresol	Renal	[28]
L-acetylcarnitine	Hepatic and renal metabolism of dietary L-carnitine Gut microbiota metabolism of L-carnitine and conversion to trimethylamine-N-oxide	Renal	[29]
Butenoyl-carnitine	Poorly studied, mainly from exogenous sources Other data not available	Data not available	https://hmdb.ca/metabolites/HMDB0249460 (accessed on 23 November 2025)
Sorbitol	Endogenous source from polyol pathway Dietary sources under gut microbiota’s action	Renal Intracellular accumulation	[30,31]

## Data Availability

The data that support the findings of this study are available from the corresponding author upon reasonable request.

## Supplementary Materials

The following supporting information can be downloaded at: https://www.mdpi.com/article/10.3390/ijms262411776/s1.

## Abbreviations

The following abbreviations are used in this manuscript:

DKD	Diabetic kidney disease
T2DM	Type 2 diabetes mellitus
ACs	Acylcarnitines
UTs	Uremic toxins
PIs	Polyol pathway intermediates
AAs	Amino acids
TNF-α	Tumor necrosis factor α
eGFR	Estimated glomerular filtration rate
TGF-β	Transforming growth factor β
ILs	Interleukins
UHPLC-QTOF-ESI^+^-MS	Ultra-high-performance liquid chromatography coupled with electrospray ionization–quadrupole–time-of-flight mass spectrometry
ELISA	Enzyme-linked immunosorbent
SD	Standard deviation
ANOVA	Analysis of variance
DM	Diabetes mellitus
uACR	Urinary albumin to creatinine ratio
DR	Diabetic retinopathy
DN	Diabetic neuropathy
HbA1c	Glycated hemoglobin
Arg	Arginine
HA	Hippuric acid
IS	Indoxyl sulfate
PCS	P-cresyl sulfate
LAC	L-acetylcarnitine
BCA	Butenoyl carnitine
Sorb	Sorbitol
Cr	Creatinine
ADMA	Asymmetric dimethylarginine
