# Supplementary material for: Gut-Derived Metabolomic Biomarkers as Mediators of the Inflammatory Pathway in Early Diabetic Kidney Disease
Source: Int J Mol Sci. 2025 Dec 5;26(24):11776. doi: 10.3390/ijms262411776 (PMC12732614; doi:10.3390/ijms262411776)
Supplement: Supplementary file 1 [file ijms-26-11776-s001.zip › 4.. Supplementary Table S1.pdf]

Table S1 Serum univariable analysis

	Independent variable	R^2	Coef β	P value
sArg	sTNF-α	0.2376	-0.1124	<0.0001
	sTGF-β	0.0960	-0.8116	0.001
	sIL-6	0.1710	-0.2206	<0.0001
	sIL-8	0.2468	-0.0562	<0.0001
	sIL-10	0.2060	1.0548	<0.0001
	sIL-12	0.1392	-0.0306	0.0001
	sIL-17	0.1367	-0.0236	0.0001
	sIL-18	0.2063	-0.0340	0.0081
sHA	sTNF-α	0.0033	0.0045	0.5504
	sTGF-β	0.0596	-0.2183	0.0105
	sIL-6	0.0036	0.0109	0.5335
	sIL-8	0.0479	-0.0084	0.0222
	sIL-10	0.0446	0.1675	0.0275
	sIL-12	0.0591	-0.0068	0.0108
	sIL-17	0.0761	-0.0060	0.0037
	sIL-18	0.0164	-0.0058	0.1851
sIS	sTNF-α	0.0845	0.0194	0.0022
	sTGF-β	0.0660	0.1947	0.0070
	sIL-6	0.0747	0.0421	0.0040
	sIL-8	0.0785	0.0091	0.0032
	sIL-10	0.0425	0.1385	0.0315
	sIL-12	0.0448	0.0050	0.0272
	sIL-17	0.0890	0.0055	0.0016
	sIL-18	0.0161	0.0049	0.1879
sLAC	sTNF-α	0.1052	0.0145	0.0006
	sTGF-β	0.0111	0.0535	0.2754
	sIL-6	0.0828	0.0298	0.0024
	sIL-8	0.0198	0.0030	0.1447
	sIL-10	0.0129	-0.0511	0.2405
	sIL-12	0.0046	0.0010	0.4846

	sIL-17	0.0125	0.0013	0.2462
	sIL-18	0.0010	0.0008	0.7380
sBCA	sTNF-α	0.3065	0.0051	<0.0001
	sTGF-β	0.1043	0.0338	0.0006
	sIL-6	0.2334	0.0103	<0.0001
	sIL-8	0.1526	0.0017	<0.0001
	sIL-10	0.1252	-0.0328	0.0002
	sIL-12	0.0899	0.0009	0.0015
	sIL-17	0.1121	0.0008	0.0004
	sIL-18	0.0720	0.0014	0.0048
sSorb	sTNF-α	0.2145	0.0039	<0.0001
	sTGF-β	0.0983	0.0302	0.0009
	sIL-6	0.1665	0.0080	<0.0001
	sIL-8	0.0945	0.0012	0.0011
	sIL-10	0.0477	0.0009	0.0573
	sIL-12	0.0407	0.0006	0.0354
	sIL-17	0.0809	0.0006	0.0027
	sIL-18	0.0334	0.0009	0.0573
