# Supplementary material for: Gut-Derived Metabolomic Biomarkers as Mediators of the Inflammatory Pathway in Early Diabetic Kidney Disease
Source: Int J Mol Sci. 2025 Dec 5;26(24):11776. doi: 10.3390/ijms262411776 (PMC12732614; doi:10.3390/ijms262411776)
Supplement: Supplementary file 1 [file ijms-26-11776-s001.zip › 5.. Supplementary Table S2.pdf]

Table S2 Urine univariable analysis

	Independent variable	R^2	Coef β	P value
uArg	uTNF-α	0.1143	0.0380	0.0003
	uTGF-β	0.0114	0.352	0.2691
	uIL-6	0.0692	0.1293	0.0057
	uIL-8	0.0068	0.0035	0.3928
	uIL-10	0.0005	-0.0145	0.8173
	uIL-12	0.0091	0.0038	0.3231
	uIL-17	0.0065	0.0015	0.4044
	uIL-18	0.0087	0.0046	0.3352
	uFABP	0.0509	0.0773	0.0184
uHA	uTNF-α	0.1240	0.6434	0.0002
	uTGF-β	0.0064	1.6415	0.4090
	uIL-6	0.0575	1.9131	0.0120
	uIL-8	0.0259	0.1115	0.0946
	uIL-10	0.0018	-0.4477	0.6602
	uIL-12	0.0147	0.0798	0.2088
	uIL-17	0.0202	0.0443	0.01407
	uIL-18	0.0148	0.0975	0.2078
	uFABP	0.0442	1.1696	0.0283
uIS	uTNF-α	0.3614	0.0316	0.0000
	uTGF-β	0.1052	0.1921	0.1052
	uIL-6	0.2938	0.1246	0.0000
	uIL-8	0.1759	0.0083	0.0000
	uIL-10	0.1576	-0.1203	0.0000
	uIL-12	0.1777	0.0080	0.0000
	uIL-17	0.1734	0.0037	0.0000
	uIL-18	0.1321	0.0084	0.0001
	uFABP	0.2543	0.0809	0.0000
uLAC	uTNF-α	0.2376	0.0072	0.0000
	uTGF-β	0.0745	0.0457	0.0041
	uIL-6	0.1782	0.0274	0.0000

	uIL-8	0.1102	0.0018	0.0004
	uIL-10	0.0511	-0.0193	0.0181
	uIL-12	0.1028	0.0017	0.0007
	uIL-17	0.0695	0.0006	0.0056
	uIL-18	0.0663	0.0016	0.0069
	uFABP	0.1782	0.0274	0.0000
uBCA	uTNF-α	0.3450	0.0064	0.0000
	uTGF-β	0.0956	0.0384	0.0011
	uIL-6	0.2711	0.0251	0.0000
	uIL-8	0.1547	0.0016	0.0000
	uIL-10	0.1230	-0.0223	0.0002
	uIL-12	0.1546	0.0015	0.0000
	uIL-17	0.1515	0.0007	0.0000
	uIL-18	0.1155	0.0016	0.0003
	uFABP	0.2411	0.0165	0.0000
uPCS	uTNF-α	0.2690	0.1047	0.0000
	uTGF-β	0.0980	0.7108	0.0009
	uIL-6	0.2273	0.4203	0.0000
	uIL-8	0.1304	0.0276	0.0001
	uIL-10	0.0743	-0.3167	0.0041
	uIL-12	0.1395	0.0271	0.0001
	uIL-17	0.1008	0.0109	0.0008
	uIL-18	0.1017	0.0282	0.0007
	uFABP	0.1975	0.2733	0.0000
